# A novel fluorescent sensor for diammonium and metal ions based on a supramolecular charge-transfer complex of bis(aza-18-crown-6)-containing dienone

**DOI:** 10.3389/fchem.2023.1263440

**Published:** 2023-10-03

**Authors:** Sergey P. Gromov, Marina V. Fomina, Ilia P. Zdorovenko, Artem N. Fakhrutdinov, Evgeny N. Ushakov

**Affiliations:** ^1^ Photochemistry Center of RAS, FSRC “Crystallography and Photonics”, Russian Academy of Sciences, Moscow, Russia; ^2^ Chemistry Department, M. V. Lomonosov Moscow State University, Moscow, Russia; ^3^ N. D. Zelinsky Institute of Organic Chemistry, Russian Academy of Sciences, Moscow, Russia; ^4^ Federal Research Center of Problems of Chemical Physics and Medicinal Chemistry, Russian Academy of Sciences, Chernogolovka, Russia

**Keywords:** dienones, charge-transfer complexes, fluorescent sensor, NMR spectroscopy, electronic spectroscopy, quantum chemical calculations

## Abstract

A bis(aza-18-crown-6)-containing 2,5-di(benzylidene)cyclopentanone and a bis(ammoniopropyl) derivative of 1,2-di(4-pyridyl)ethylene in MeCN were found to form a supramolecular charge-transfer complex, which can act as an “off–on” fluorescent sensor for the Ca^2+^ and 1,12-dodecanediammonium ions. The molecular structure of this complex in solution was studied by density functional theory calculations.

## 1 Introduction

Luminescent molecular sensors based on crown compounds are widely used to detect cations (De Silva et al., 1997; Ushakov et al., 2010; Li et al., 2017; Jiang et al., 2020; Golcs et al., 2021). The general concept of the design of these sensors is based mainly on the phenomena of changes in luminescence due to intramolecular processes in donor–acceptor systems, primarily such as photoinduced electron transfer and the transition of an excited molecule to a twisted state with internal charge transfer. The macrocyclic and luminophore units of the “off-on” luminescent molecular sensors should be connected in such a way that the substrate entering the macrocycle cavity be able to suppress the process responsible for luminescence quenching.

Organic charge-transfer complexes (Foster, 1969) attract attention of researchers owing to their wide use in various fields of materials science and chemistry such as development of organic solar cells (Günes et al., 2007; Huo et al., 2019), electromagnetic materials (Wu et al., 2021), organic semiconductors (Morita et al., 2013), photocatalysts (Yuan et al., 2020), and fluorescent sensors (Gromov et al., 2008; Vedernikov et al., 2011; Yao et al., 2017; Shakya and Khan, 2021), as model systems for studying ultrafast electron transfer reactions (Ushakov et al., 2004), and for the design of artificial light-harvesting antennas (Ke et al., 2017; Lian et al., 2020).

Molecular systems consisting of two or more structurally organized components that perform a common specific function are classified as molecular devices (Lehn, 1995; Balzani et al., 2003). Separately, each component (fragment) of the system perform only its particular action, but as a result of the cooperation of components, a more complex function arises. The molecular devices in which non-covalent interactions are used to combine components can be designated as supramolecular devices. Promising molecules for use as building blocks of supramolecular devices are biscrown-containing unsaturated compounds (Fery-Forgues and Al-Ali, 2004), which, in addition to the ability to form host–guest complexes between macrocyclic fragments and metal ions, also exhibit fluorescent properties due to the presence of an ethylene unit in the chromophore moiety. We have found that the formation of hydrogen bonds between a biscrown compound of this type and a π-electron acceptor molecule can provide not only a very high thermodynamic stability of the donor–acceptor complex, but also the spatial preorganization of the components for efficient charge-transfer interaction (Gromov et al., 2008). Therefore, two-component systems of this type can be attributed to such a class of photoactive supramolecular devices as supramolecular fluorescent sensors (Lehn, 1995; MakoRacicot and Levine, 2019).

**SCHEME 1:**
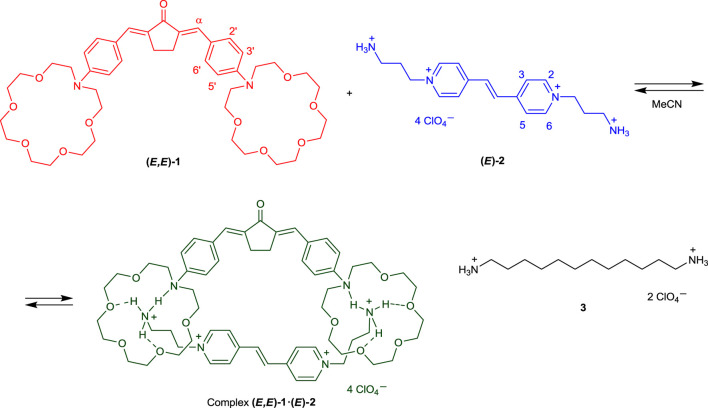
Chemical structure of compounds **1**–**3** and formation of supramolecular pseudocyclic complex **1∙2**.

In this communication, we present the results of experimental and theoretical studies of the supramolecular donor–acceptor complex between bis(azacrown)-containing dienone **1** (π-electron donor) and viologen analog, bis(ammoniopropyl) derivative of dipyridylethylene **2** (π-electron acceptor, Scheme 1). The complex is formed via hydrogen bonding and is characterized by intermolecular charge transfer in the ground state. Presumably, complex **1∙2** has a pseudocyclic structure (Scheme 1). Salt **3** was used in this study as the model diammonium compound containing no π-electron system.

## 2 Materials and methods

### 2.1 Materials

MeCN (extra high purity, water content <0.03%, Cryochrom) was used to prepare solutions.

Compounds **1**, **2** and 1,12-dodecanediammonium diperchlorate (compound **3**) were prepared according to known procedures (refs: Fomina et al., 2020; Gromov et al., 1999; Gromov et al., 2009, respectively).

Complex [**1∙2**] was obtained as a dark red oil by mixing equimolar amounts of **1** and **2** in a minimum volume of acetonitrile. After evaporation of the solvent, the residue was washed with benzene and dried.

The stoichiometry of complex [**1∙2**] was confirmed by ^1^H NMR, 2D DOSY NMR (in MeCN-*d*_3_), IR (ATR), and elemental analysis data. Dark-red oil (14.9 mg, 97% yield); ^1^HNMR (500.13 MHz, MeCN-*d*_3_, 25°C) δ 1.31–1.37 (br.m, 4H), 2.77 (br.s, 4H), 2.78–2.83 (br.m, 4H), 3.51 (m, 8H), 3.68–3.72 (br.m, 22H), 3.76 (m, 24H), 4.20–4.24 (m, 4H), 7.08–7.13 (br.m, 6H), 7.33 (m, 4H), 7.70 (br.s, 2H), 8.15 (d, J = 6.7 Hz, 4H), 8.43 (d, J = 6.7 Hz, 4H); IR (ATR): 1,633 cm^−1^ (C=O), 3,125 cm^−1^ (N^+^−H). Analysis (calcd., found for C_43_H_62_N_2_O_11_·C_19_H_30_C_l4_N_4_O_16_·2H_2_O): C (48.29, 47.99), H (6.24, 6.19) N (5.54, 5.61).

### 2.2 Methods

The ^1^H NMR spectra were recorded on Bruker Avance-500 and Bruker Avance-III 400 spectrometers (operating frequencies of 500.13 and 400.13 MHz, respectively) in MeCN-*d*_3_ at 25°C using the solvent signal as the internal standard (δ_H_ 1.96 ppm). The chemical shifts were determined with an accuracy of 0.01 ppm, and the spin–spin coupling constants were determined with an accuracy of 0.1 Hz. The NMR spectra were processed by TopSpin 3.2 software and diffusion coefficients were calculated using T1/T2 relaxation module.

IR spectra were recorded using a Nicolet iS5 Fourier transform spectrometer (Thermo Scientific) with an internal reflectance attachment and a diamond optical element for attenuated total reflectance (ATR, iD7) with 45° angle of incidence. The resolution was 4 cm^−1^, and the number of scans was 20.

UV-vis absorption spectra were measured on a Cary 4000 spectrophotometer in MeCN. Uncorrected fluorescence spectra were obtained on a Cary Eclipse spectrofluorimeter at room temperature. All manipulations with solutions of compounds **1** and **2** were performed in a darkroom under red light.

Fluorescence quantum yield measurements were carried out on a time-resolved fluorescence spectrometer FluoTime 300 (PicoQuant), equipped with a PDL-820 multichannel picosecond laser driver and an LDH-P-C-440 semiconductor pulsed laser as the excitation source (*λ* = 440 nm, pulse duration ∼80 ps).

Fluorescence quantum yields (*φ*) were determined using fluorescein in 0.1 N NaOH in water as the reference standard (*φ*_*ref*_ = 0.92, Rurack, 2008) by the following equation:

$$\varphi_{sample}=\varphi_{ref}\times \frac{I_{sample}}{I_{ref}}\times \frac{D_{ref}}{D_{sample}}\times \frac{n_{sample}^{2}}{n_{ref}^{2}}$$

where *I* is the integral intensity of the corrected fluorescence spectrum, *D* is the optical density of the solution at the excitation wavelength, *n* is the refractive index of the solvent.

The measured quantum yields are as follows: dienone **1**, *φ* = 0.16; a mixture of **1** and **2** in 1:8 ratio, *φ* = 0.01; the 1:8 mixture after the addition of diammonium salt **3** in 15-fold excess over **1**, *φ* = 0.18; the 1:8 mixture after the addition of Ca(ClO_4_)_2_ in 30-fold excess over **1**, *φ* = 0.09.

### 2.3 Computational details

Molecular mechanics was used to generate sets of low-energy conformations for compound **1** and complex **1∙2**. Conformational searches were carried out using the MMFF94s force field (Halgren, 1999). The subsequent calculations by density functional theory (DFT) and time-dependent DFT (TDDFT) were performed using the Gaussian 16 program package (Frisch et al., 2016). The molecular mechanics geometries were re-optimized using the M06-2X density functional (Zhao and Truhlar, 2008) with the 6-31G(d) basis set (Hehre et al., 1972; Hariharan and Pople, 1973). The universal solvation model known as Solution Model Density (SMD) (Marenich et al., 2009) was employed to account for solvent effects. The default optimization criteria were tightened 3-fold using the internal option IOp (1/7 = 100). The geometry optimizations were followed by harmonic frequency calculations in order to verify the nature of stationary points and to derive thermochemical quantities. The thermochemical analysis was carried out using a scale factor of 0.9678 for harmonic frequencies (Kesharwani et al., 2015). The Gibbs free energy in solution (*G*_soln_) was calculated as follows:

$$G_{\text{soln}}=E_{\text{soln}}+\Delta G_{\text{corr}}$$

where *E*_soln_ is the electronic energy in solution and Δ*G*_corr_ is the thermal correction to *G*_soln_, including the zero-point energy. To minimize basis set superposition errors for complexes, the *E*_soln_ values were derived from single-point calculations with the larger 6-311G(2df,2p) basis set (Krishnan et al., 1980; Frisch et al., 1984). The calculated *G*_soln_ values were used to identify the most stable conformation for each molecular structure.

TDDFT calculations were carried out on the M06-2X/6-31G(d)/SMD geometries using the CAM-B3LYP functional (Yanai et al., 2004), the 6-311G(2d,p) basis set, and the SMD solvation model. This approach has been previously used to study the electronic transitions in a supramolecular charge-transfer complex involving bis(18-crown-6)azobenzene (Ushakov et al., 2020). It was shown that in the case of CAM-B3LYP, the energies of electronic transitions are usually significantly overestimated, but the positions of electronic transitions of different nature relative to each other are predicted quite well.

The natural transition orbitals (Martin, 2003) were derived from the TDDFT calculations as described in the Gaussian manual. The orbitals were visualized using the Chemcraft program (Zhurko, 2005).

## 3 Results and discussion

### 3.1 Electronic absorption and fluorescence spectroscopy

**FIGURE 1:**
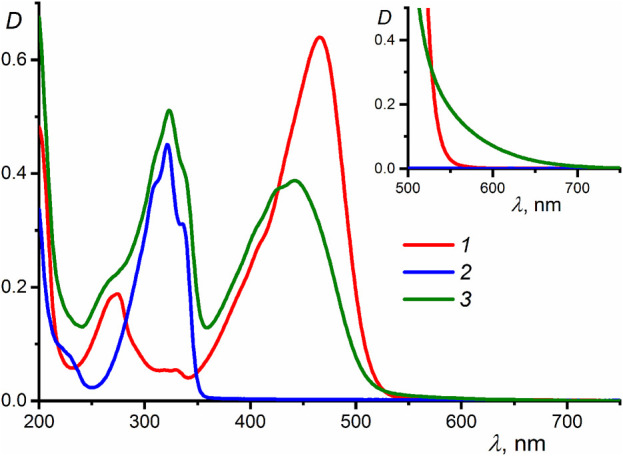
UV-vis absorption spectra in MeCN: (1) dienone **1**, (2) diammonium compound **2**, and (3) equimolar mixture of **1** and **2** (reactant concentrations of 1 × 10^−5^ M); the inset shows the spectra of the same systems but measured at reactant concentrations of 2 × 10^−4^ M.

Figure 1 shows the UV-vis absorption spectra of compounds **1** (*λ*_max_ = 466 nm, *ε*_max_ = 64,000 mol^−1^dm^3^cm^−1^), **2** (*λ*_max_ = 321 nm, *ε*_max_ = 44,000 mol^−1^dm^3^cm^−1^), and their equimolar mixture in MeCN, recorded at two different concentrations of the reactants. The fact that the absorption spectrum of the mixture measured at a low concentration of **1** and **2** (1 × 10^−5^ M) differs considerably from the sum of the spectra of separate components is indicative of the formation of a strong complex between **1** and **2**. The complex formation induces a blue shift and a decrease in the intensity of the long-wavelength absorption band of **1** (*λ*_max_ = 442 nm, *ε*_max_ = 39,000 mol^−1^dm^3^cm^−1^), which is related to the electronic transition with charge transfer from the electron-donating phenylazacrown ether moieties to the electron-withdrawing cyclopentanone center. The increase in the energy of this transition is attributable to the decrease in the electron-donating effect of the phenylazacrown ether moieties due to hydrogen bonding with the ammonium groups of **2** (Scheme 1).

A characteristic feature of the formation of organic donor–acceptor complexes with intermolecular charge transfer is the appearance of a new, low-intensity band (charge-transfer band) in the long-wavelength region of the UV-vis spectrum, which is absent in the spectra of the free donor and acceptor (Foster, 1969; Gromov et al., 2008). The absorption spectra of compounds **1** and **2** and their equimolar mixture measured at high component concentrations (2 × 10^−4^ M, see the inset in Figure 1) indicate that the formation of complex **1∙2**, apart from inducing a blue shift of the dienone intense band, results in increased absorbance at the long-wavelength edge of the spectral curve. This fact can be due to the appearance of a weak, low-energy electronic transition characterized by intermolecular charge transfer. Parameters of the charge-transfer absorption band such as the peak position and molar absorptivity cannot be determined, as this band is superimposed by a broad intense band associated with a local transition in the dienone.

**FIGURE 2:**
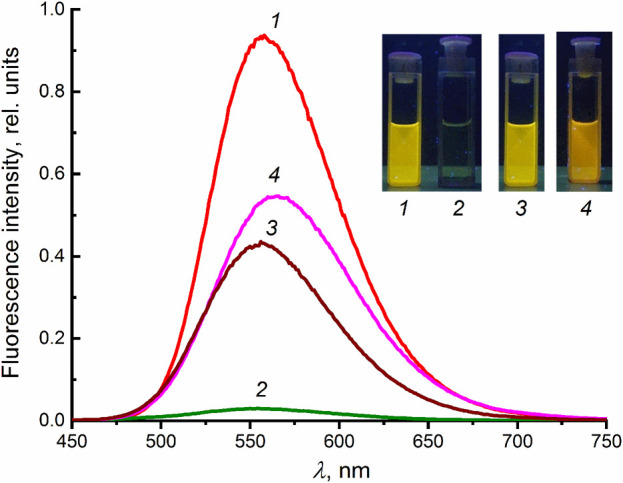
Uncorrected fluorescence spectra in MeCN (excitation at 428 nm): (1) dienone **1** (4 × 10^−6^ M), (2) a mixture of **1** and **2** in 1:8 ratio, (3) the 1:8 mixture in the presence of diammonium salt **3** in a concentration of 6 × 10^−5^ M, and (4) the 1:8 mixture in the presence of Ca(ClO_4_)_2_ in a concentration of 1.2 × 10^−4^ M.

Figure 2 shows the fluorescence spectra of dienone **1** (*λ*_max_ = 556 nm) and its mixture with **2** in MeCN. The formation of complex **1∙2** leads to almost complete quenching of fluorescence of **1**, which supports the hypothesized charge-transfer nature of this complex. Previously, we studied the complex formation of **1** with a number of diammonium cations NH_3_^+^(CH_2_)_*n*_NH_3_^+^ with polymethylene chain length *n* = 2–12 (Fomina et al., 2020). A pseudocyclic complex was found to form in the case of the 1,12-dodecanediammonium ion **3**. Good geometric matching of the components owing to the fact the distance between the ammonium groups in **3** is close to the distance between the binding sites in **1** provides rigid structure of complex **1∙3**, which results in an increase in the fluorescence intensity of **1**. Considering the geometric matching of the components of complex **1∙2**, one could expect an increase in the fluorescence intensity of **1** upon complex formation. However, contrary to the expectations, the formation of complex **1∙2** leads to fluorescence quenching. The addition of excess diammonium salt **3** or calcium perchlorate to a solution of **1∙2** induces a fluorescence enhancement due to the destruction of the charge-transfer complex.

The fluorescence quantum yields of the studied compounds are presented in Section 2.2. The main spectroscopic parameters (absorption and fluorescence) are given in Supplementary Table S1 (Supplementary Material).

### 3.2 NMR spectroscopy studies

**FIGURE 3:**
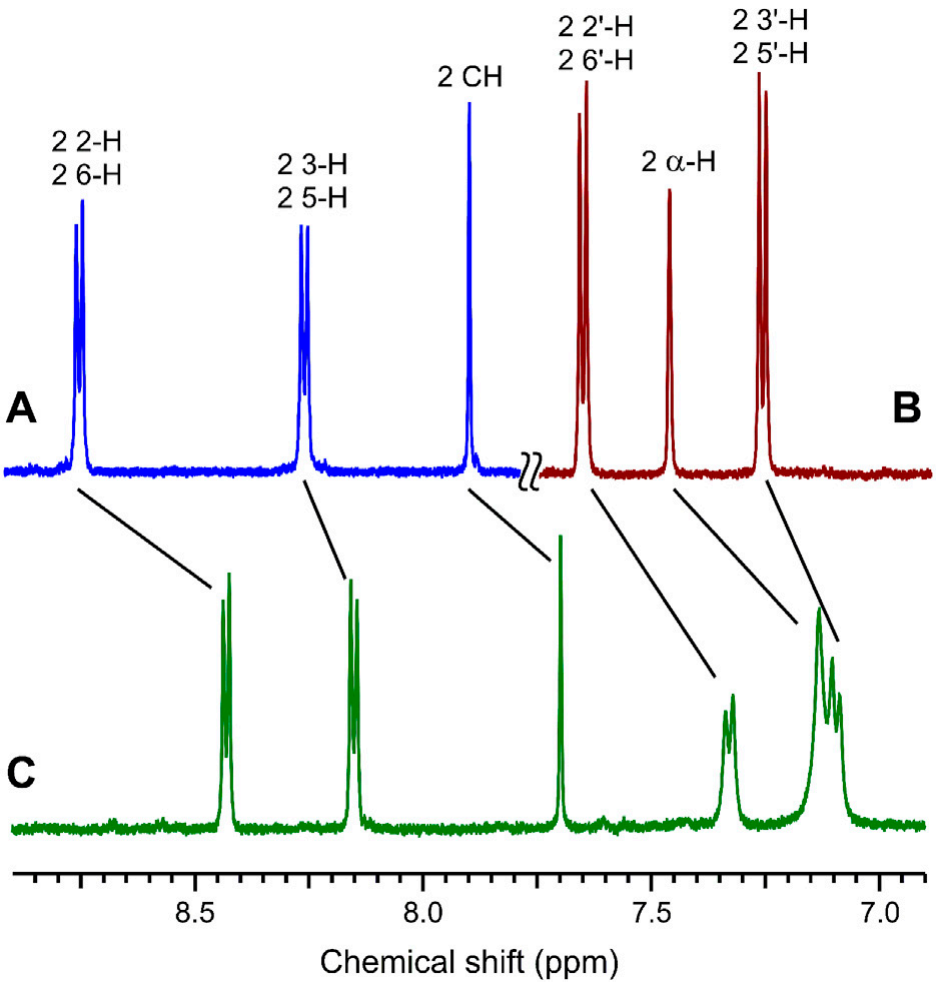
^1^H NMR spectra (aromatic proton region) in MeCN-*d*_3_ at 25°C: **(A)** compound **2**, **(B)** equimolar mixture of dienone **1** and diammonium salt **3**, and **(C)** equimolar mixture of **1** and **2**; the reactant concentrations are 1 × 10^−3^ M.

The complex formation of dienone **1** with compounds **2** and **3** induces pronounced changes in the chemical shifts in the ^1^H NMR spectra (Figure 3). In a mixture of dienone **1** and dipyridylethylene derivative **2**, most aliphatic and aromatic proton signals of **1** and **2** are shifted upfield, while in a mixture of dienone **1** with diammonium salt **3**, most proton signals of compound **1** are shifted downfield. The most pronounced changes in the chemical shifts (0.11–0.38 ppm) are observed for an equimolar mixture of **1** and **2**, which attests to the double-decker structure of complex **1∙2**. In this complex, the central moieties of the components are located close to each other, which is responsible for the upfield shift of the signals of most protons due to mutual shielding.

More information about the structure of complex **1** with dipyridylethylene derivative **2** can be derived from the 2D NOESY spectrum of a saturated solution of a mixture of these compounds in MeCN-*d*_*3*_ (Supplementary Figure S6, Supplementary Material).

**FIGURE 4:**
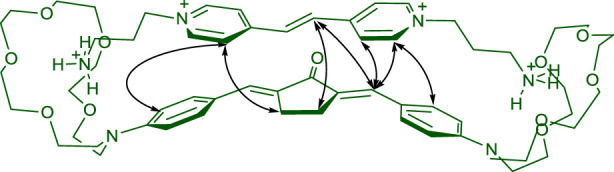
Intermolecular NOE interactions for complex **1·2**.

Figure 4 schematically shows intermolecular NOE interactions found for complex **1∙2**. Apart from the intense intramolecular cross-peaks, there are peaks corresponding to the intermolecular through-space interaction of the ethylene protons and the pyridine ring protons of viologen analogue **2** with the 3-CH_2_ and 4-CH_2_ protons of the cyclopentanone ring, α-H protons of the ethylene bonds, and the 2′-H, 3′-H, 5′-H, and 6′-H benzene protons of dienone **1**. All of the detected intermolecular NOE cross-peaks have low intensity compared to that of the intramolecular NOE interactions; therefore, the distances between the interacting protons can be roughly estimated to be in the 3.0–3.5 Å range.

Considering these interactions, we may propose the most probable structure for the complex of dienone **1** with dipyridylethylene derivative **2**, which is shown in Scheme 1. The ammonium groups of viologen analogue **2** are bound simultaneously to two azacrown ether residues of dienone **1**, and the pyridine rings and the ethylene unit of dipyridylethylene derivative **2** are located above (or near) the central moiety of molecule **1**. In other words, the complex of dienone **1** with dipyridylethylene derivative **2** is a pseudocyclic structure.

The diffusion ordered NMR spectroscopy (DOSY), a sensitive method capable of distinguishing the spectra of single compounds in a mixture, is widely used in the host–guest chemistry (Gafni and Cohen, 1997; Frish et al., 1999; Frish et al., 2000; Avram, and Cohen, 2002; Cohen et al., 2005; Specht et al., 2005; Gulino et al., 2006). Compounds **1** and **2** have different diffusion coefficients (1.07^−9^ m^2^/s and 9.49^−10^ m^2^/s, respectively); a mixture of **1** and **2** shows only one diffusion coefficient (9.12^−10^ m^2^/s), which is lower than the diffusion coefficients of single **1** or **2**, thus confirming the presence of a common discrete supramolecular system, complex **1·2** (the 2D DOSY spectra of **1**, **2**, and their equimolecular mixture are shown in Supplementary Figure S7 (Supplementary Material). Thus, analysis of ^1^H NMR and 2D DOSY spectra, in combination with elemental analysis data, indicates the formation of a strong supramolecular complex.

### 3.3 Quantum chemical calculations

The molecular structures of dienone **1** and complex **1∙2** in MeCN were calculated using density functional theory (DFT), and the nature of the low-energy electronic transitions in these two structures was studied by time-dependent DFT (TDDFT). Details of the DFT and TDDFT calculations are given in Section 2.3, and the Cartesian coordinates for all atoms in the calculated structures are presented in the Supplementary Material (Supplementary Tables S2, S3).

Supplementary Figure S8 (Supplementary Material) shows the calculated structures of **1** and **1∙2**. According to calculations, the most stable conformer of free dienone **1** in MeCN is a C_2_-symmetrical structure. The geometry of the dibenzylidenecyclopentanone residue of **1** is slightly non-planar; the maximal torsion angle in the cyclopentanone ring is 9.9°, and the torsion angles between the C=C bonds and the benzene rings are 8.4°.

The most stable conformer of complex **1∙2** in MeCN is an asymmetric pseudocyclic structure in which the dibenzylidenecyclopentanone residue of **1** and the dipyridylethylene residue of **2** are located one above the other. The complex formation leads to a significant distortion of the geometry of the dibenzylidenecyclopentanone residue; in the complex, this fragment looks noticeably bent and faces the dipyridylethylene residue of **2** with its convex side. The torsion angles between the C=C bonds and the benzene rings in the dibenzylidenecyclopentanone residue are 14.6° and −17.1°, and the maximal torsion angle in the cyclopentanone ring is 8.7°. In the dipyridylethylene residue of **2**, the torsion angles between the C=C bond and the pyridinium rings are as low as 2.2° and 3.1°. In the calculated structure **1∙2**, the intermolecular distances between the hydrogen atoms of the benzylidenecyclopentanone residue of **1** and the dipyridylethylene residue of **2** are consistent with the NOESY spectroscopy data (Section 3.2).

Table 1 shows the dominant natural transition orbital (NTO) pairs and their occupation numbers for the first excited singlet state of **1** and the first three excited singlet states of **1∙2**, as derived from TDDFT calculations. It can be seen that the S_0_-S_1_ transition in free dienone **1** is accompanied by a significant charge redistribution: the electron density on the aniline residues decreases, while that in the central part of the molecule increases. The first two excited states of complex **1∙2** are characterized by intermolecular charge transfer, i.e., in both cases, excitation leads to the transition of an electron from an orbital located mainly on the donor moiety (dienone **1**) to an orbital localized on the acceptor moiety (compound **2**). The third excited state of **1∙2** results from a local electronic transition in the dienone moiety; this state is similar in nature to the S_1_ state of free dienone **1**. Note that all the NTO pairs discussed here are characterized by high occupation numbers (0.92–0.98).

**TABLE 1:** Dominant natural transition orbital pairs and their occupation numbers for low-lying excited singlet states of dienone 1 and complex 1∙2 in MeCN; hydrogen atoms in structure 1∙2 are not shown except for those of the ammonium groups.

Excited state	Dominant NTO pairs		Occupation number
	Hole	Particle	
S_1_ (**1**)	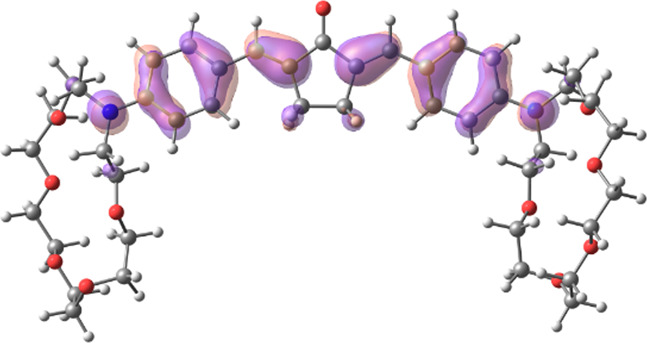	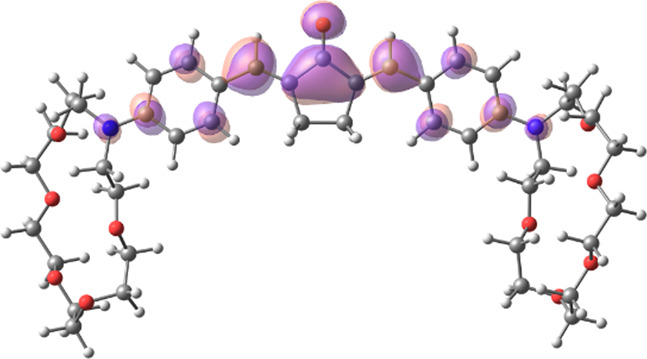	0.9380
S_1_ (**1∙2**)	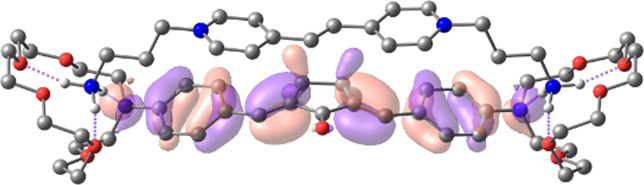	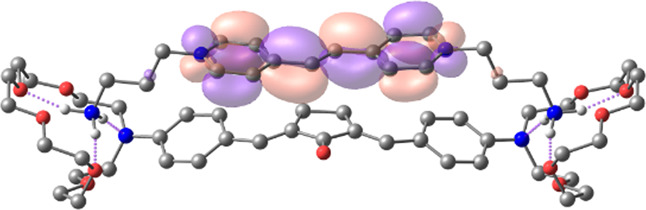	0.9776
S_2_ (**1∙2**)	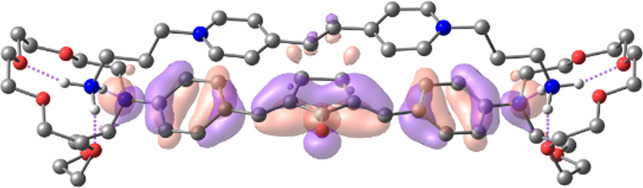	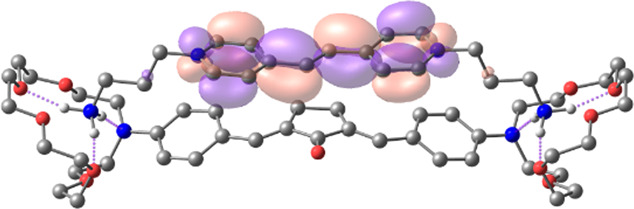	0.9556
S_3_ (**1∙2**)	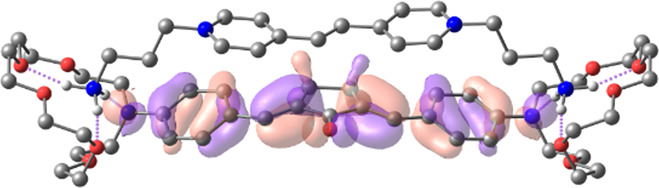	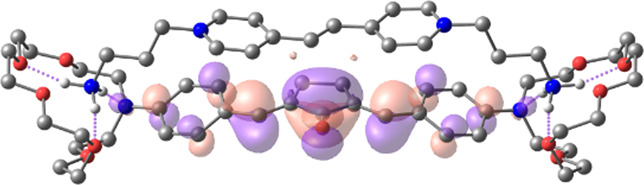	0.9235
	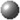 – carbon, 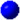 – nitrogen, 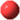 – oxygen, 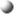 – hydrogen		

Table 2 presents the TDDFT-calculated energies and oscillator strengths for low-energy electronic transitions in dienone **1** and complex **1∙2**. At a qualitative level, the theoretical results agree with the UV-vis absorption spectroscopy data (Section 3.1). First, the calculations predict a significant blue shift and a decrease in the intensity of the long-wavelength absorption band of dienone **1** upon the complexation with compound **2**. Second, the weak S_0_-S_1_ transition in complex **1∙2** was calculated to occur at a lower energy than the intense S_0_-S_1_ transition in free dienone **1**, which can explain the fact that the complex formation leads to some increase in the absorbance at the long-wavelength edge of the absorption spectrum of **1** (Section 3.1, the inset in Figure 1).

**TABLE 2:** Parameters of low-energy electronic transitions in dienone 1 and complex 1∙2, as calculated by TDDFT.

Compound	Transition	Energy, eV	Oscillator strength
**1**	S_0_-S_1_	3.1166	2.090
**1**	S_0_-S_2_	3.5426	0.008
**1∙2**	S_0_-S_1_	2.8962	0.023
**1∙2**	S_0_-S_2_	3.1981	0.072
**1∙2**	S_0_-S_3_	3.3292	1.352

## 4 Conclusion

A novel donor–acceptor supramolecular system was developed, involving bis(azacrown)-containing dienone **1** and unsaturated viologen analog **2**. The results of experimental and theoretical studies show that the pseudocyclic complex between **1** and **2**, characterized by intra-supramolecular charge transfer, is a promising example of a supramolecular system capable of producing a strong fluorescence response upon the interaction with the Ca^2+^ and 1,12-dodecanediammonium ions. The results suggest the possibility of using complexes of this type as such photoactive supramolecular devices as supramolecular fluorescent sensors.

## Data Availability

The original contributions presented in the study are included in the article/Supplementary Material, further inquiries can be directed to the corresponding author.
