# Supplementary material for: A novel fluorescent sensor for diammonium and metal ions based on a supramolecular charge-transfer complex of bis(aza-18-crown-6)-containing dienone
Source: Front Chem. 2023 Oct 3;11:1263440. doi: 10.3389/fchem.2023.1263440 (PMC10579611; doi:10.3389/fchem.2023.1263440)
Supplement: Supplementary file 1 [file DataSheet1.PDF]

A Novel Fluorescent Sensor for Diammonium and Metal Ions Based on a Supramolecular Charge-Transfer Complex of Bis(aza-18-crown-6)-containing Dienone

Sergey P. Gromov*[1,2], Marina V. Fomina[1], Ilia P. Zdorovenko[1,2], Artem N. Fakhrutdinov[3], Evgeny N. Ushakov[1,4]

[1]Photochemistry Center of RAS, FSRC "Crystallography and Photonics", Russian Academy of Sciences, Moscow, Russian Federation.

[2]Chemistry Department, M. V. Lomonosov Moscow State University, Moscow, Russian Federation.

[3]N. D. Zelinsky Institute of Organic Chemistry, Russian Academy of Sciences, Moscow, Russian Federation.

[4]Federal Research Center of Problems of Chemical Physics and Medicinal Chemistry, Russian Academy of Sciences, Chernogolovka, Russian Federation.

* **Correspondence**: Sergey P. Gromov: spgromov@mail.ru

CONTENT:

1 The ^{1}H NMR spectra of compounds **1**, **2**, a mixture of compound **1** and **2**, a mixture of compound **1** and **3** (Figures S1-S5) 2

2 The NOESY NMR spectra of a mixture of compound **1** and **2** (Figure S6) 4

3 The DOSY NMR spectra of compounds **1**, **2** and a mixture of compound **1** and **2** (Figure S7) 5

4 Spectroscopy data for compounds **1**, **2**, a mixture of **1** and **2**, a mixture of **1** and **2** in the presence of diammonium salt **3** and a mixture of **1** and **2** in the presence of $Ca(ClO_4)_2$ (Table S1) 7

5 Structures of dienone **1** and complex **1**·**2** in MeCN, as calculated by DFT (Figure S8) 8

6 Cartesian coordinates for all atoms in the DFT calculated structures of dienone 1 and complex **1**·**2** in MeCN (Tables S2 and S3) 8

1 The ^{1}H NMR spectra of compounds 1, 2, a mixture of compound 1 and 2, a mixture of compound 1 and 3

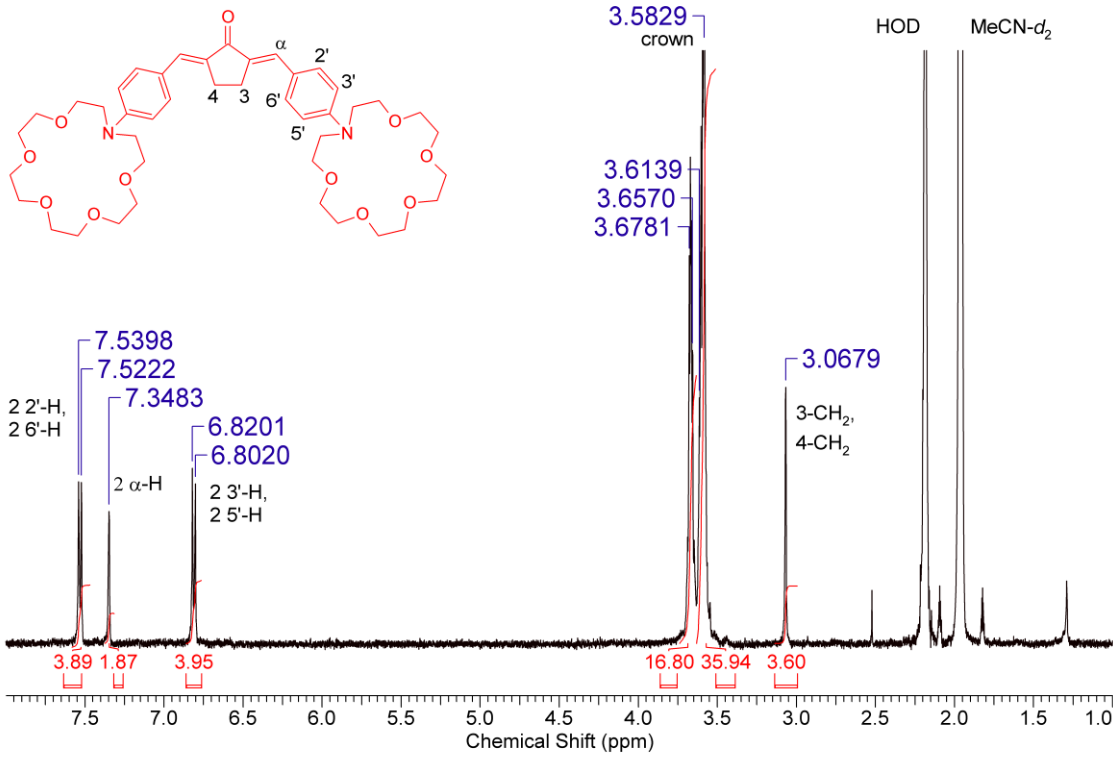

Figure S1. ^{1}H NMR spectrum of compound **1**; MeCN-d_3, 25 °C.

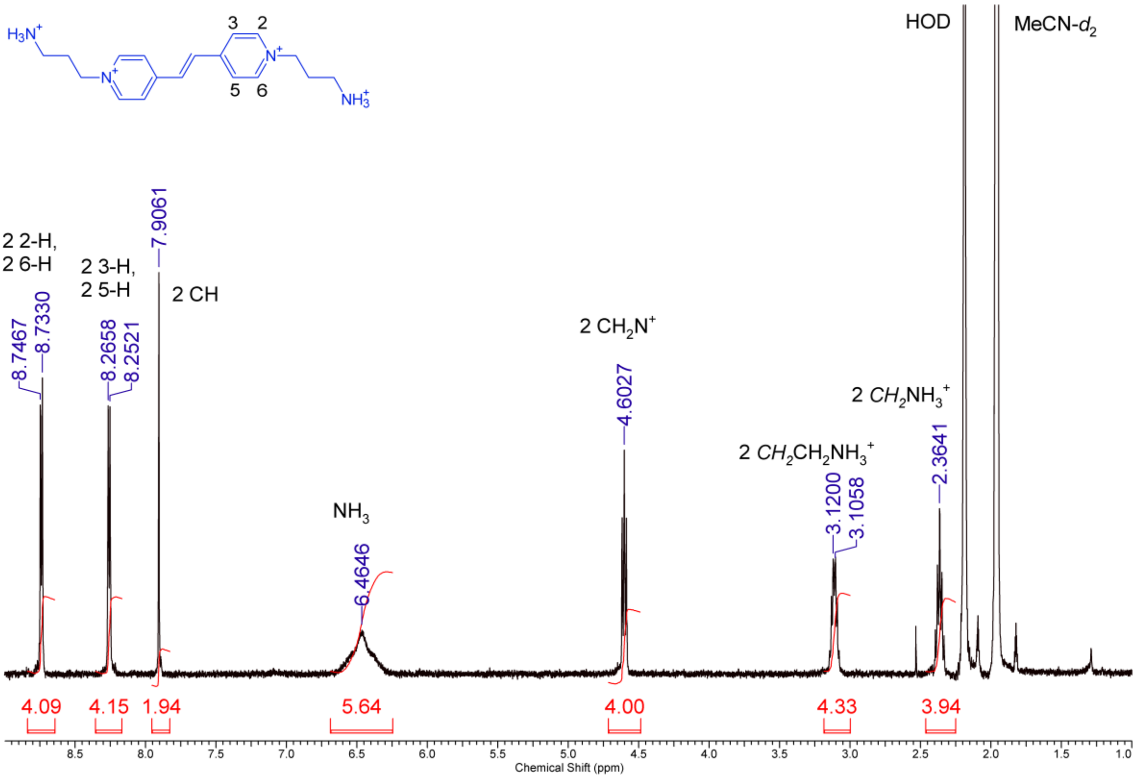

Figure S2. ^{1}H NMR spectrum of compound **2**; MeCN-d_3, 25 °C.

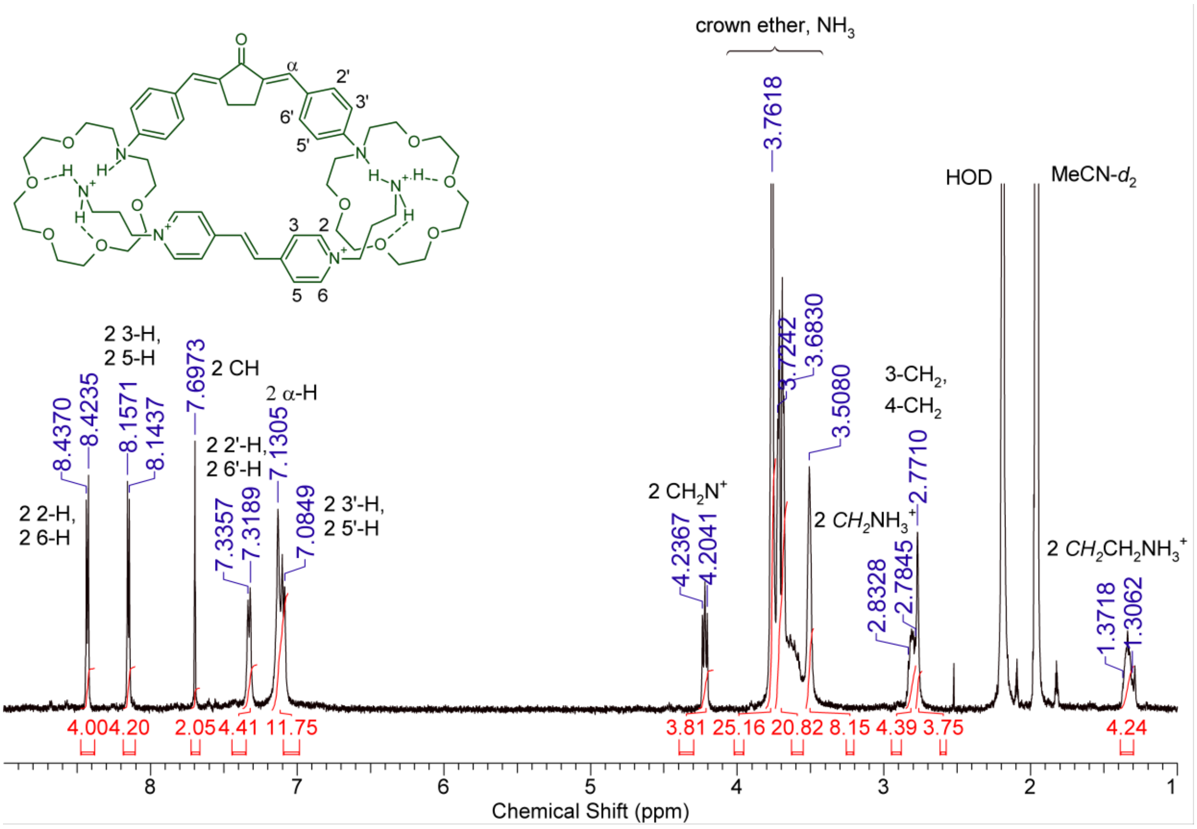

Figure S3. ^{1}H NMR spectrum an equimolar mixture of **1** and **2**; the reactant concentrations are 1×10^{-3} M; MeCN-d_3, 25 °C.

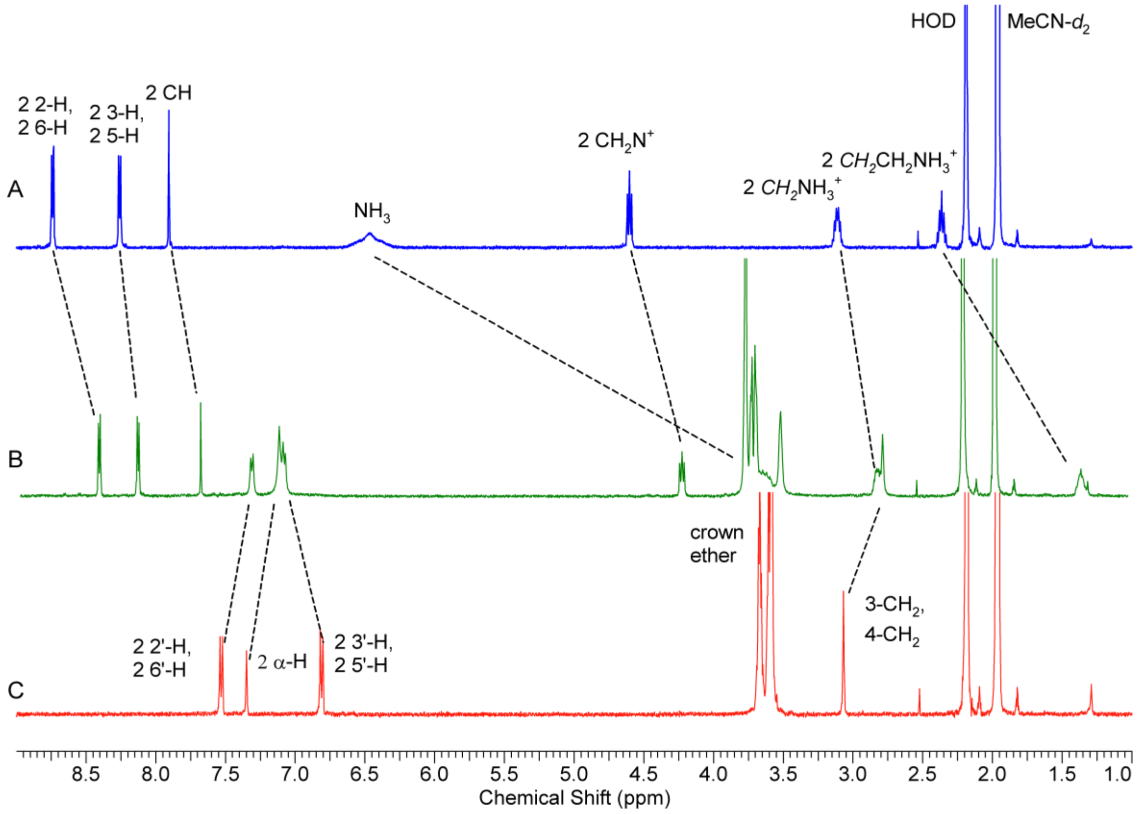

Figure S4. ^{1}H NMR spectra in MeCN-d_3 at 25 °C: (**A**) compound **2**, (**B**) equimolar mixture of dienone **1** and **2**, and (**C**) compaund **1**; the reactant concentrations are 1×10^{-3} M.

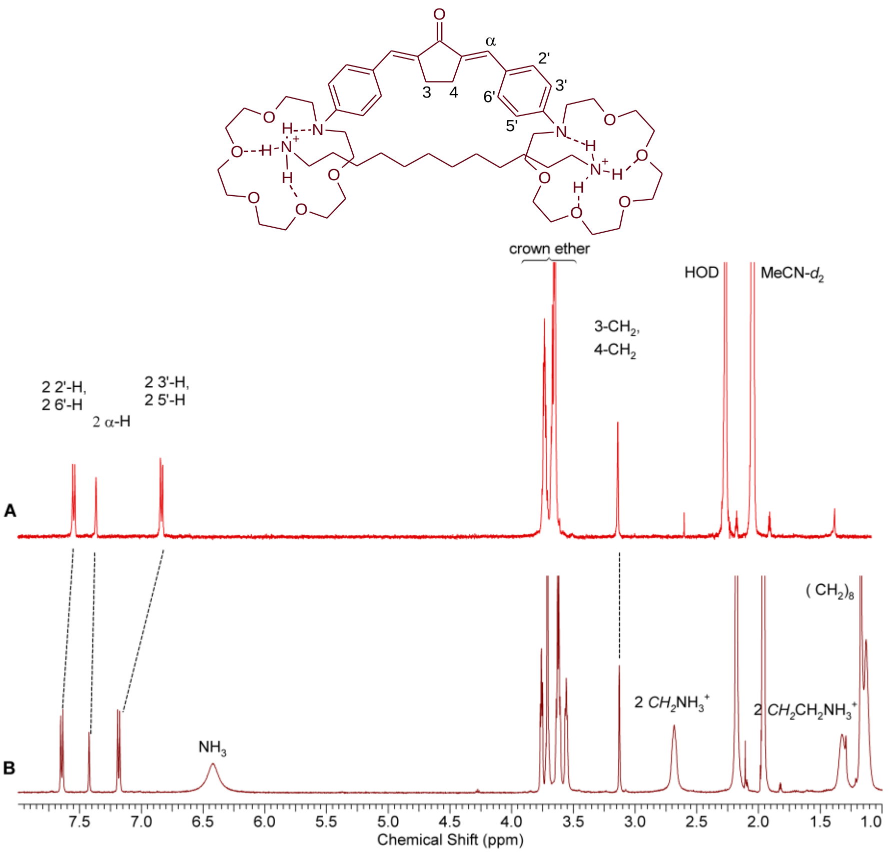

Figure S5. ^{1}H NMR spectra in MeCN-d_3 at 25 °C: (**A**) compound **1** and (**B**) equimolar mixture of dienone **1** and **3**; the reactant concentrations are $1{\times}10^{-3}$ M.

2 The NOESY NMR spectra of a mixture of compound 1 and 2

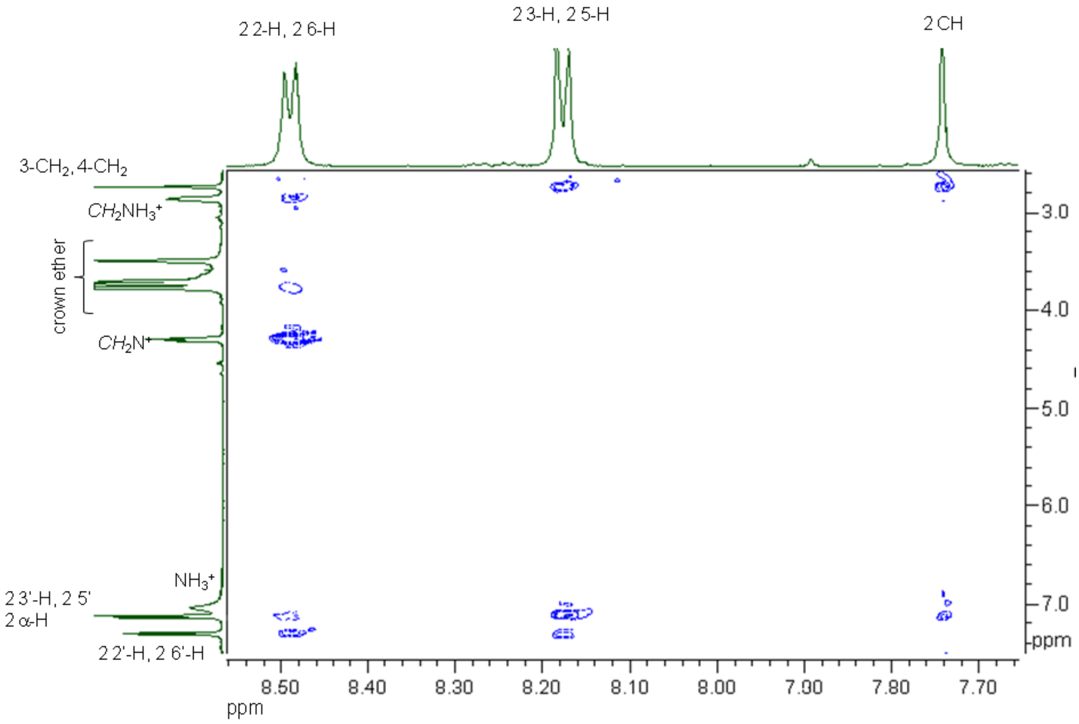

Figure S6. Fragment of the NOESY spectrum of a mixture of compound **1** and viologen analog **2**, MeCN-d_3, 25 °C, the reactant concentrations are $2.4{\times}10^{-3}$ M.

3 The DOSY NMR spectra of compounds 1, 2 and a mixture of compound 1 and 2

A

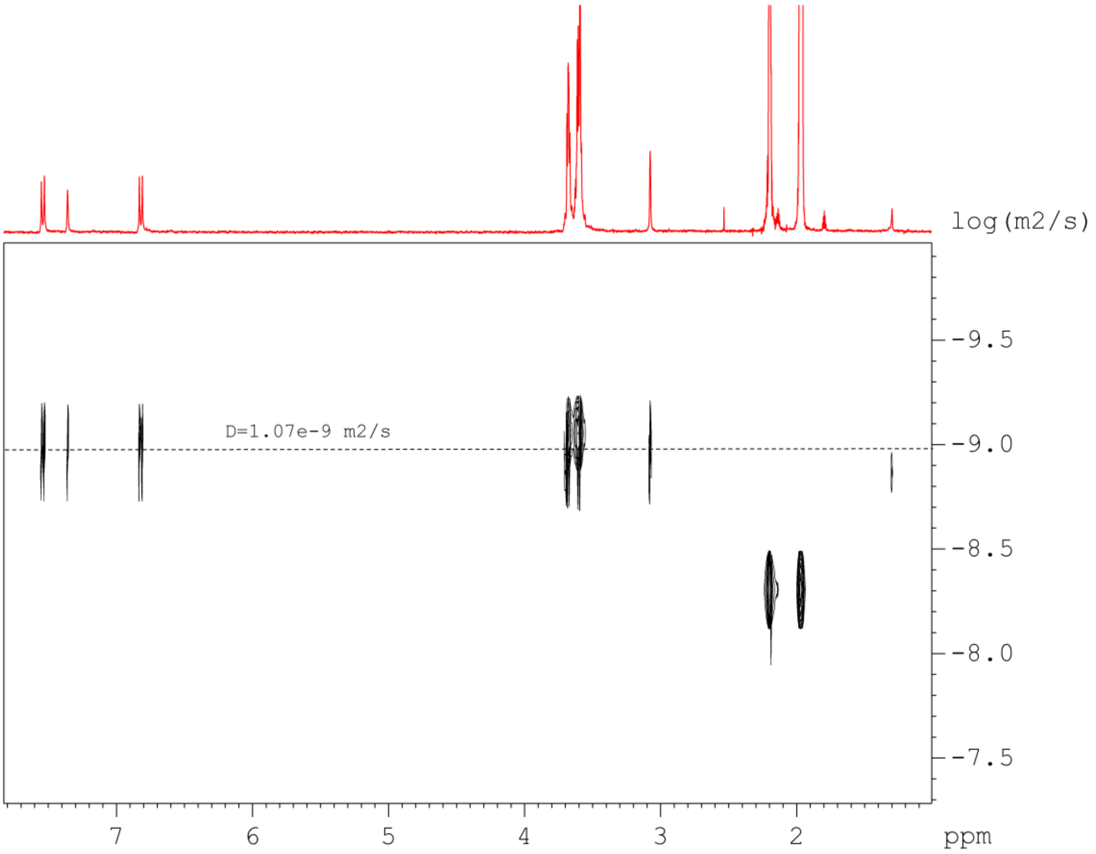

B

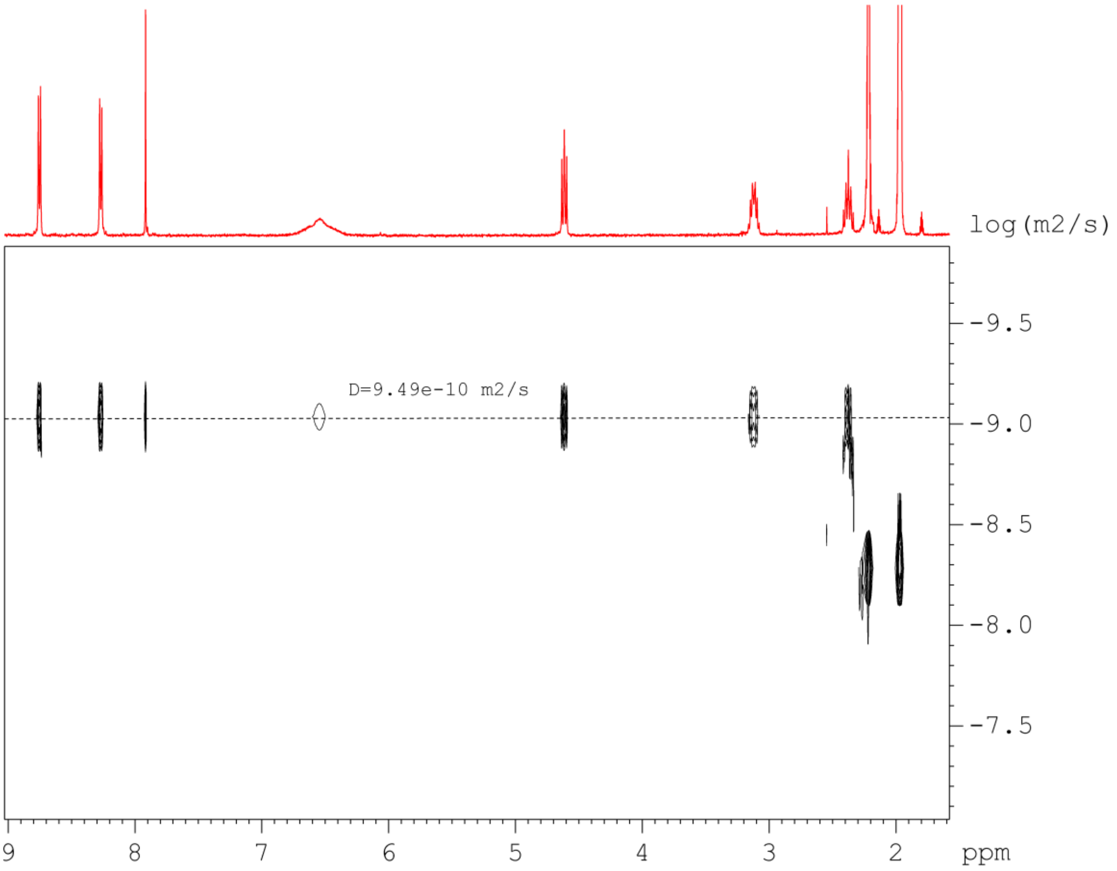

C

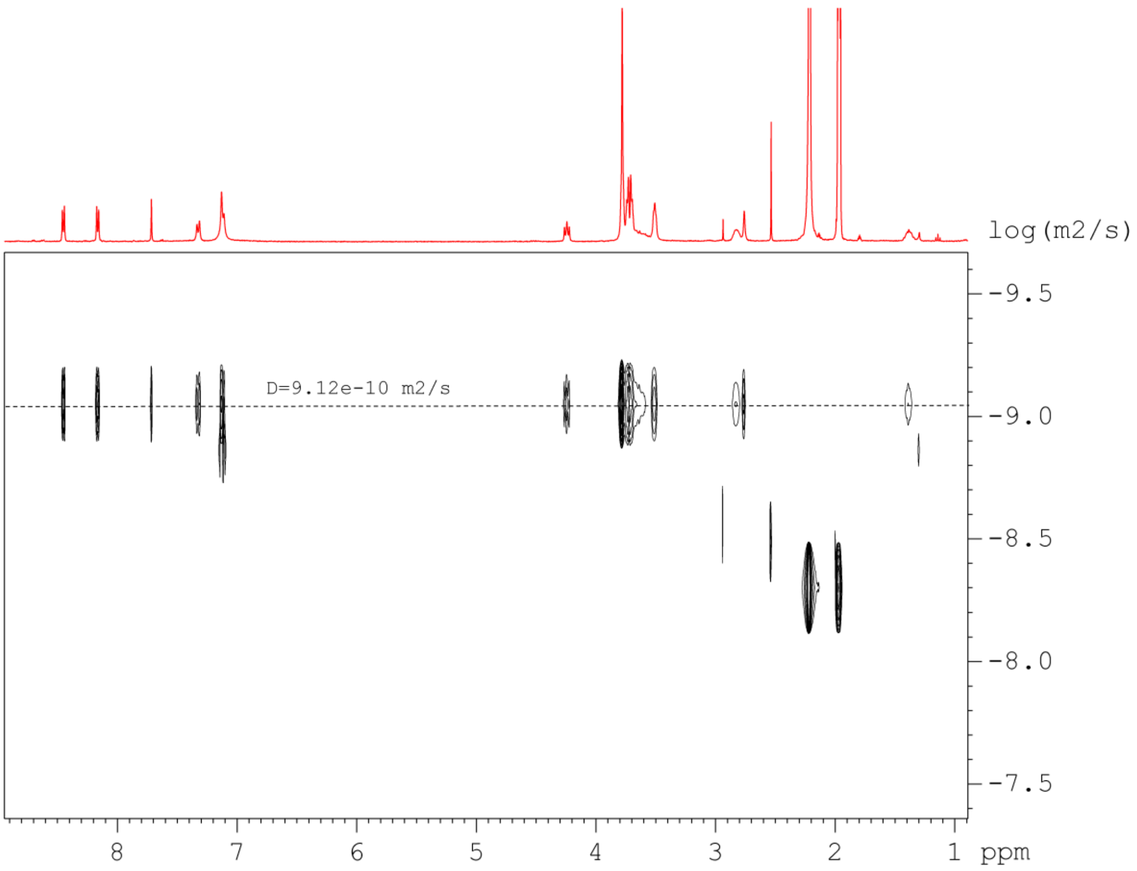

Figure S7. 2D DOSY NMR spectra: (**A**) **1** (C_1 = 3×10^{-3} M), (**B**) **2** (C_2 = 3×10^{-3} M), (**C**) equimolar mixture of **1** and **2**; 500 MHz, MeCN-d_3, 25 °C.

4 Spectroscopy data for compounds 1, 2, a mixture of 1 and 2, a mixture of 1 and 2 in the presence of diammonium salt 3 and a mixture of 1 and 2 in the presence of $Ca(ClO_4)_2$ (Table S1)

Table S1. Spectroscopy data for compounds **1**, **2**, a mixture of **1** and **2**, a mixture of **1** and **2** in the presence of diammonium salt **3** and a mixture of **1** and **2** in the presence of $Ca(ClO_4)_2$[a]

Compound	λ_{max}^{abs} (nm)	ε_{max} ($mol^{-1}dm^3cm^{-1}$)	λ_{max}^{em} (nm)[b] (λ_{ex} 440 nm)	φ_f^c (λ_{ex} 440 nm)
dienone **1** (1×10^{-5} M)	466	64000	568	0.16
diammonium compound **2** (1×10^{-5} M)	321	44000	-	-
equimolar mixture of **1** and **2** (reactant concentrations of 1×10^{-5} M)	442	39000	-	-
a mixture of **1** (6×10^{-7} M) and **2** in 1:8 ratio	-	-	568	0.01
the 1:8 mixture in the presence of diammonium salt **3** (9×10^{-6} M)	-	-	562	0.18
the 1:8 mixture in the presence of $Ca(ClO_4)_2$ (1.8×10^{-5} M)			572	0.09

[a] In MeCN at ambient temperature.
[b] The corrected fluorescence spectrum.
[c] The fluorescence quantum yields derived from the corrected fluorescence spectra.

5 Structures of dienone 1 and complex 1·2 in MeCN, as calculated by DFT

A

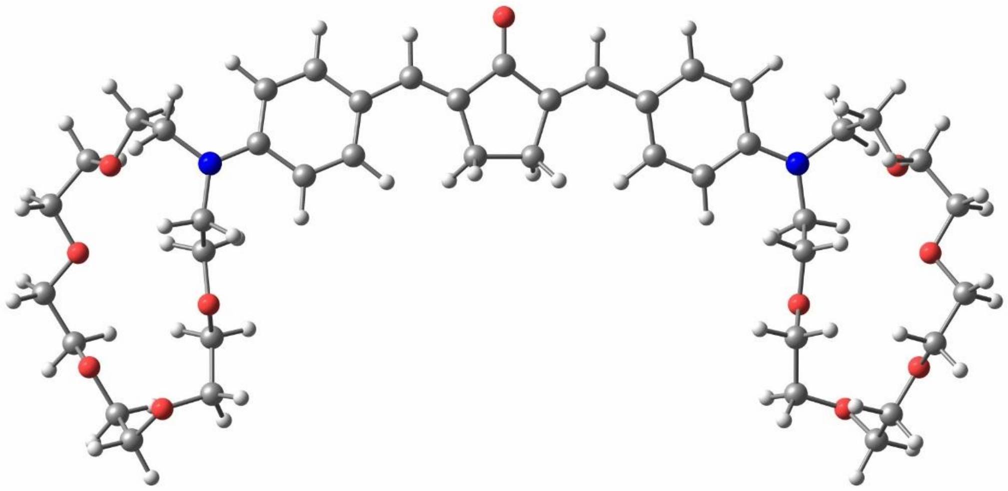

B

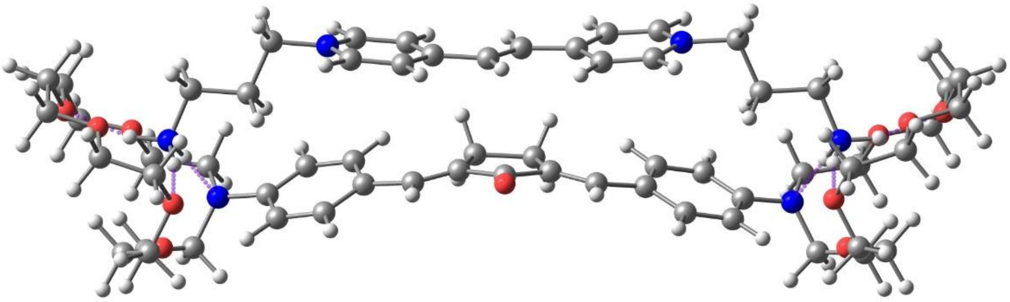

Figure S8. Structures of dienone **1** (**A**) and complex **1·2** (**B**) in MeCN, as calculated by DFT.

6 Cartesian coordinates for all atoms in the DFT calculated structures of dienone 1 and complex 1·2 in MeCN (Tables S1 and S2).

Table S2. Cartesian coordinates for all atoms in the DFT calculated structure of dienone **1** (the first column is atomic numbers; the next three columns are coordinates in Å).

6	0.130539	2.422874	3.703355
1	0.208833	2.421216	4.792145
6	0.023524	1.200731	3.143406
6	0.000000	-0.000000	4.017105
6	-0.023524	-1.200731	3.143406
6	0.065682	-0.773932	1.700265
1	-0.715960	-1.238910	1.090539
1	1.025938	-1.074768	1.264984
6	-0.065682	0.773932	1.700265
1	-1.025938	1.074768	1.264984
1	0.715960	1.238910	1.090539
8	0.000000	-0.000000	5.242102
6	-0.130539	-2.422874	3.703355
1	-0.208833	-2.421216	4.792145
8	2.603093	9.643522	1.467792

8	4.499494	9.944026	-0.747762
8	3.934011	9.812871	-3.649898
8	1.542311	8.577809	-4.706293
8	1.086852	7.485852	-2.056222
7	0.318170	7.677182	1.518705
6	0.173485	3.740149	3.089052
6	0.466749	4.851171	3.899359
6	0.538062	6.138485	3.398435
6	0.293294	6.400332	2.028044
6	-0.000292	5.282467	1.206738
1	-0.190799	5.414825	0.148246
6	-0.060579	4.001178	1.726015
6	0.566216	8.819039	2.384521
1	0.047564	8.677853	3.337015
1	0.128164	9.702493	1.911655
6	2.039050	9.093093	2.640005
6	3.996506	9.826141	1.598233
6	4.521608	10.668809	0.462966
6	5.150589	10.664047	-1.773645
6	5.204869	9.844661	-3.038661
6	3.960691	9.064884	-4.843833
6	2.592875	9.090197	-5.497637
6	1.696685	7.222998	-4.328340
6	2.155582	7.092043	-2.889844
6	1.448980	7.512063	-0.694229
1	2.253281	8.238007	-0.510242
1	1.806818	6.520572	-0.372235
6	0.197436	7.904018	0.088012
1	0.655849	4.692146	4.958635
1	0.792832	6.942172	4.078299
1	-0.311445	3.192127	1.049413
1	2.145136	9.801075	3.477002
1	2.559246	8.163111	2.917406
1	4.513332	8.854422	1.615986
1	4.221297	10.346050	2.542281
1	3.920395	11.586988	0.376092
1	5.554422	10.965748	0.704201
1	6.180576	10.902663	-1.465900
1	4.631318	11.614386	-1.971918
1	5.941002	10.296685	-3.721592
1	5.547216	8.824723	-2.802270
1	4.281558	8.030797	-4.638397
1	4.687320	9.493402	-5.553117
1	2.319433	10.126174	-5.720182
1	2.664140	8.540778	-6.449578
1	2.395437	6.701352	-4.996304
1	0.716615	6.741009	-4.425489
1	2.436006	6.046482	-2.681020
1	3.031420	7.731694	-2.708408
1	-0.011227	8.964554	-0.068618
1	-0.657550	7.347302	-0.312910
8	-2.603093	-9.643522	1.467792

8	-4.499494	-9.944025	-0.747762
8	-3.934011	-9.812871	-3.649898
8	-1.542311	-8.577809	-4.706293
8	-1.086852	-7.485852	-2.056222
7	-0.318170	-7.677182	1.518705
6	-0.173485	-3.740149	3.089052
6	-0.466749	-4.851171	3.899359
6	-0.538062	-6.138485	3.398435
6	-0.293294	-6.400332	2.028044
6	0.000292	-5.282467	1.206738
1	0.190799	-5.414825	0.148246
6	0.060579	-4.001178	1.726015
6	-0.566216	-8.819039	2.384521
1	-0.047564	-8.677853	3.337015
1	-0.128164	-9.702493	1.911655
6	-2.039050	-9.093093	2.640005
6	-3.996506	-9.826141	1.598233
6	-4.521608	-10.668809	0.462966
6	-5.150589	-10.664047	-1.773645
6	-5.204869	-9.844661	-3.038661
6	-3.960691	-9.064884	-4.843833
6	-2.592875	-9.090197	-5.497637
6	-1.696685	-7.222998	-4.328340
6	-2.155582	-7.092043	-2.889844
6	-1.448980	-7.512063	-0.694229
1	-2.253281	-8.238007	-0.510242
1	-1.806818	-6.520572	-0.372235
6	-0.197436	-7.904018	0.088012
1	-0.655849	-4.692146	4.958635
1	-0.792832	-6.942172	4.078299
1	0.311445	-3.192127	1.049413
1	-2.145136	-9.801075	3.477002
1	-2.559246	-8.163111	2.917406
1	-4.513332	-8.854422	1.615986
1	-4.221297	-10.346050	2.542281
1	-3.920396	-11.586988	0.376092
1	-5.554422	-10.965748	0.704201
1	-6.180576	-10.902662	-1.465900
1	-4.631318	-11.614386	-1.971918
1	-5.941002	-10.296685	-3.721592
1	-5.547216	-8.824723	-2.802270
1	-4.281558	-8.030797	-4.638397
1	-4.687320	-9.493402	-5.553117
1	-2.319433	-10.126174	-5.720182
1	-2.664140	-8.540778	-6.449578
1	-2.395437	-6.701352	-4.996304
1	-0.716615	-6.741009	-4.425489
1	-2.436006	-6.046482	-2.681020
1	-3.031420	-7.731694	-2.708408
1	0.011227	-8.964554	-0.068618
1	0.657550	-7.347302	-0.312910

Table S3. Cartesian coordinates for all atoms in the DFT calculated structure of complex **1·2** (the first column is atomic numbers; the next three columns are coordinates in Å).

8	9.427300	1.026700	2.325200
8	7.753500	1.758100	4.411200
8	8.747900	-0.037600	6.358700
8	10.356900	-2.083900	5.341500
8	9.956300	-3.010200	2.696000
7	8.588900	-1.349200	0.757500
6	5.455200	0.612500	0.604000
1	5.108800	1.600200	0.899000
6	6.782300	0.275800	0.803600
1	7.457900	0.983400	1.274300
6	7.260000	-1.001600	0.448000
6	6.351700	-1.910000	-0.122800
1	6.673400	-2.898100	-0.429100
6	9.640200	-0.367200	0.428700
1	9.196800	0.463500	-0.127300
1	10.374500	-0.841000	-0.232700
6	10.350400	0.198300	1.648200
1	11.222700	0.784700	1.324800
1	10.701800	-0.605500	2.314900
6	9.955200	1.707900	3.445700
1	10.813600	2.329300	3.152800
1	10.288500	0.990500	4.214300
6	8.838600	2.577100	3.980400
1	9.205300	3.208000	4.796800
1	8.471900	3.227900	3.178700
6	7.434500	1.861300	5.792300
1	6.516900	1.281100	5.929200
1	7.230000	2.906100	6.059100
6	8.524100	1.317900	6.690200
1	9.456200	1.890300	6.570800
1	8.206000	1.414100	7.739100
6	9.808900	-0.592300	7.110500
1	9.589400	-0.539900	8.186700
1	10.738400	-0.035500	6.920900
6	9.983900	-2.033200	6.710200
1	10.769100	-2.488300	7.328900
1	9.050700	-2.593300	6.868400
6	10.663600	-3.406900	4.930000
1	9.790000	-4.059600	5.070400
1	11.491100	-3.801600	5.534900
6	11.070300	-3.392200	3.480200
1	11.904300	-2.691300	3.326200
1	11.410000	-4.398700	3.197000
6	10.224200	-3.131600	1.312800
1	11.093600	-2.517200	1.037100
1	10.469300	-4.174500	1.065500
6	8.979200	-2.743700	0.541500
1	8.168300	-3.384900	0.896400
1	9.143500	-2.954700	-0.526200

6	5.020000	-1.559000	-0.318400
6	4.537800	-0.287600	0.030600
1	4.352200	-2.298100	-0.747300
6	-0.848600	0.388600	-2.854400
1	-1.160100	1.286300	-2.317500
6	0.240500	-0.227100	-2.356800
6	0.937300	0.369100	-1.185000
6	2.232500	-0.345100	-1.020700
6	2.271300	-1.532900	-1.945800
1	2.251800	-2.469400	-1.373800
1	3.190900	-1.547100	-2.540500
6	1.010300	-1.426300	-2.847100
1	0.411900	-2.342600	-2.778100
1	1.282700	-1.310400	-3.902500
8	0.536300	1.301300	-0.502800
6	3.170100	0.166600	-0.201200
1	2.887500	1.083300	0.319400
8	-5.681000	1.900900	-7.385200
8	-6.827900	2.466600	-4.928900
8	-9.107400	0.814000	-5.180600
8	-8.961900	-1.037600	-7.271000
8	-6.436400	-2.043100	-8.086000
7	-4.024500	-0.552800	-7.502300
6	-2.504800	1.064200	-4.542200
1	-2.592100	2.006400	-4.006400
6	-3.251900	0.869500	-5.690100
1	-3.933200	1.641800	-6.033600
6	-3.176600	-0.347100	-6.396900
6	-2.309300	-1.340600	-5.910000
1	-2.199900	-2.284800	-6.430200
6	-4.111600	0.532200	-8.499100
1	-3.383300	1.309300	-8.250500
1	-3.835400	0.135500	-9.482600
6	-5.486600	1.175600	-8.582200
1	-5.522500	1.852300	-9.448200
1	-6.276700	0.417100	-8.702300
6	-6.883100	2.641800	-7.326600
1	-6.945500	3.348100	-8.166800
1	-7.755900	1.969000	-7.368000
6	-6.853600	3.392700	-6.013500
1	-7.715500	4.063800	-5.940800
1	-5.940300	3.996000	-5.963300
6	-7.937900	2.544600	-4.044600
1	-7.705900	1.866000	-3.218000
1	-8.035700	3.561700	-3.643800
6	-9.240500	2.134900	-4.696500
1	-9.497000	2.812200	-5.524800
1	-10.048900	2.197400	-3.952900
6	-10.260500	0.392900	-5.881800
1	-11.144200	0.424700	-5.228500
1	-10.443400	1.054300	-6.741500
6	-10.047900	-1.020200	-6.356700

1	-10.960400	-1.376900	-6.853200
1	-9.835000	-1.683800	-5.505800
6	-8.786300	-2.322300	-7.848000
1	-8.597400	-3.068000	-7.062300
1	-9.697100	-2.613300	-8.388800
6	-7.628700	-2.281300	-8.810100
1	-7.784400	-1.490600	-9.559000
1	-7.570200	-3.245500	-9.335000
6	-5.294200	-2.150200	-8.913500
1	-5.368700	-1.450600	-9.758700
1	-5.225400	-3.164200	-9.333000
6	-4.058800	-1.901000	-8.072500
1	-4.078100	-2.619200	-7.248600
1	-3.166800	-2.102900	-8.685000
6	-1.561700	-1.131600	-4.755800
6	-1.635300	0.076200	-4.043400
1	-0.912700	-1.930800	-4.415600
6	7.078200	-1.890800	4.323800
6	5.791600	-1.636300	3.552900
6	4.647400	-2.461500	4.124100
6	2.611700	-1.245200	3.531300
6	1.490500	-1.046600	2.755200
6	2.095300	-2.974700	1.459200
6	3.206500	-3.123200	2.259700
7	3.438600	-2.281000	3.287800
7	8.170700	-1.048400	3.777000
1	6.977400	-1.634300	5.380900
1	7.394300	-2.933600	4.235600
1	5.534100	-0.570500	3.593900
1	5.931500	-1.908700	2.500700
1	4.389000	-2.156300	5.139300
1	4.882700	-3.527100	4.124300
1	2.874400	-0.605400	4.366100
1	0.836000	-0.212000	2.980000
1	1.940800	-3.693100	0.662600
1	3.928100	-3.919800	2.120200
1	7.975400	-0.038400	3.905900
1	9.053300	-1.266100	4.275700
1	8.331500	-1.233600	2.769000
6	1.194900	-1.927400	1.705000
6	-6.685800	-1.243100	-4.728500
6	-5.414300	-1.204300	-3.895500
6	-5.528800	-2.118600	-2.683900
6	-3.856300	-1.240700	-1.131900
6	-2.641000	-1.284800	-0.488000
6	-2.147200	-3.309700	-1.677000
6	-3.376500	-3.217200	-2.296900
7	-4.216400	-2.207900	-2.003000
7	-6.581200	-0.276100	-5.848600
1	-7.567500	-0.963600	-4.147300
1	-6.843200	-2.233000	-5.165400
1	-5.213800	-0.177600	-3.565000

1	-4.566400	-1.531400	-4.507700
1	-6.248200	-1.743000	-1.954300
1	-5.811500	-3.133000	-2.970600
1	-4.581000	-0.451900	-0.967200
1	-2.401800	-0.487000	0.205000
1	-1.500200	-4.146500	-1.916600
1	-3.727600	-3.943800	-3.020500
1	-6.549400	0.702500	-5.508300
1	-7.413800	-0.370800	-6.458600
1	-5.744900	-0.456400	-6.435400
6	-1.760000	-2.351300	-0.731700
6	-0.481000	-2.525100	-0.036100
1	0.096300	-3.393700	-0.346600
6	-0.041900	-1.721800	0.946200
1	-0.624900	-0.857300	1.254200
